# Evaluation of Zearalenones and Their Metabolites in Chicken, Pig and Lamb Liver Samples

**DOI:** 10.3390/toxins14110782

**Published:** 2022-11-11

**Authors:** Paula Llorens Castelló, Matteo Antonio Sacco, Isabella Aquila, Juan Carlos Moltó Cortés, Cristina Juan García

**Affiliations:** 1 Laboratory of Food Chemistry and Toxicology, Faculty of Pharmacy, University of Valencia, 46100 Burjassot, Spain; 2 Institute of Legal Medicine, Department of Medical and Surgical Sciences, “Magna Graecia”, Università degli Studi “Magna Graecia” di Catanzaro, 88100 Catanzaro, Italy

**Keywords:** liver, zearalenone, zearalanone, chicken, lamb, pig

## Abstract

Zearalenone (ZON), zearalanone (ZAN) and their phase I metabolites: α-zearalenol (α-ZOL), β-zearalenol (β-ZOL), α-zearalalanol (α-ZAL) and β-zearalalanol (β-ZAL) are compounds with estrogenic activity that are metabolized and distributed by the circulatory system in animals and can access the food chain through meat products from livestock. Furthermore, biomonitoring of zearalenones in biological matrices can provide useful information to directly assess mycotoxin exposure; therefore, their metabolites may be suitable biomarkers. The aim of this study was to determine the presence of ZON, ZAN and their metabolites in alternative biological matrices, such as liver, from three different animals: chicken, pig and lamb, in order to evaluate their exposure. A solid–liquid extraction procedure coupled to a GC-MS/MS analysis was performed. The results showed that 69% of the samples were contaminated with at least one mycotoxin or metabolite at varying levels. The highest value (max. 152.62 ng/g of β-ZOL) observed, and the most contaminated livers (42%), were the chicken liver samples. However, pig liver samples presented a high incidence of ZAN (33%) and lamb liver samples presented a high incidence of α-ZOL (40%). The values indicate that there is exposure to these mycotoxins and, although the values are low (ranged to 0.11–152.6 ng/g for α-ZOL and β-ZOL, respectively), analysis and continuous monitoring are necessary to avoid exceeding the regulatory limits and to control the presence of these mycotoxins in order to protect animal and human health.

## 1. Introduction

Zearalenone (ZON) is a common non-steroidal estrogen mycotoxin that was isolated for the first time from maize contaminated by *Fusarium* genera [1]. Different fungi species such as *F. culmorum*, *F. graminearum*, *F. crookwellense*, *F. equiseti* can produce this mycotoxin [2,3]. ZON is a common and potent contaminant of cereals and grain-derived products. It has been detected worldwide in various products including corn, peas, maize, eggs, fish feed, and fibrous feed [4,5]. Corn is the main contaminated cereal and the literature reports a mean value of 14 ng/g of ZON in samples from Morocco [6], 48 ng/g in samples from Germany [7], and 9.45 ng/g in samples from Pakistan [8]. 

ZON can directly affect foodstuffs intended for mammals. Maximum standards levels have been assessed by Commission Regulation (EC) No. 1881/2006 and Commission Recommendation No. 2006/576/EC, i.e., 0.1 ng/g in feed for piglets and 0.25 ng/g in feed for porkers [9]. Various adverse effects of ZON on mammals are reported, including endocrine and reproductive disorders but also immunotoxicity, hematotoxicity, genotoxicity, hepatotoxicity [10]. ZON-estrogenic and anabolic properties are due to the binding to estrogen receptors with high affinity. In animals that are more sensitive to this mycotoxin, ZON causes estrogenic disorders such as infertility, uterine hypertrophy, feminization, testicular atrophy, miscarriage, vaginal prolapse or breast enlargement [11,12]. In addition, ZON can cause a decrease in sperm count, and disorders of progesterone and testosterone levels. Direct hepatotoxic effects have also been assessed. Dolenšek et al. have reported increases in hepatocellular necrosis, apoptosis and inflammation of hepatic lobules in pigs fed with contaminated food [13]. For piglets or pigs, WHO has reported the lowest observed adverse effect level (LOAEL) in the range from 17.6 µg/kg bw/day to 200 µg/kg bw/day, while the no effect level (NOEL) ranges from 10.4 µg/kg bw/day to 40 µg/kg [1,14,15].

After oral exposure, ZON is rapidly absorbed in the gastrointestinal tract and then distributed in various organs with slow body elimination. The liver is the most important organ for ZON metabolism, followed by the kidneys, bowel, and reproductive organs. The metabolism of ZON occurs in the intestinal cells, involving the production of two metabolites, i.e., α-zearalenol (α-ZOL) and β-zearalenol (β-ZOL), that are produced through a reduction catalyzed by 3α- and 3β-hydroxy-5-steroid dehydrogenases (HSDs), and then α-zearalanol (α-ZAL) and β-zearalanol (β-ZAL) via double reduction. In addition, zearalanone (ZAN) is produced through a reversible reduction. The metabolites, particularly α-ZAL and α-ZOL, show the main estrogenic activity, while β-ZOL, has shown a lower estrogenic activity [1,2].

Along with mycotoxin analysis in food, biomonitoring of ZON through biological matrices can provide useful information thus directly assessing mycotoxin exposure and metabolites levels [16]. In this way, mycotoxin biomarkers, represented by ZON phase I or phase II metabolites, may be suitable candidates to acquire exposure information for biomonitoring [17,18]. Nevertheless, as there is a lack of standards for quantification of conjugates, cleavage of ZON products by using β-glucuronidase may be a proper alternative to quantify the conjugated mycotoxins. Methods for analyzing ZEN and metabolite presence in biological samples have been developed to study its exposure in animals for clinical or research purposes [19]. Kwaśniewska et al. (2015) reviewed the methodology for determination of ZEN and metabolites in tissue samples [20]. Many methods require a similar procedure of extraction, and clean-up steps prior to instrumental analysis. The most common instrumental methods capable of separating ZEN and metabolites from other compounds are liquid (LC) and gas chromatography (GC) combined with mass spectrometry (MS) to identify them by ion mass. Until now, LC methods are the most used [20,21]; however, recent studies have indicated that GC may be capable of providing similar sensitivity [22]. Methods proposed for ZON quantification in tissue samples are gas chromatography (GC), which can provide good sensitivity for ZON, and metabolites evaluation. However, this does not avoid the necessity of having optimized homogenization and extraction methods prior to its analysis [23,24]. Among biological matrices, body fluids have been widely used to measure ZON intake and risk assessment, including urine, serum, and breast milk. Studies carried out in pigs’ urine, the incidence was 100% in Croatian samples (mean ± SD 238 ± 30 µg/L) and 92% in Swedish samples (mean ± SD 2.44 ± 4.39 ng/mL) [25,26]; in pig serum, an incidence of 50% was found in Bulgarian samples (mean ± SD 0.24 ± 0.12 μg/L) and an incidence of 17.3% was found in Romanian samples (mean 0.8 ng/mL) [27,28].

Despite numerous studies focused on body fluids, few papers have measured the levels of ZON and metabolites in animal organs. The liver and kidneys represent important targets of ZON, with the possible accumulation of mycotoxins contributing to slow elimination from the body. Also, these two organs from animal origins are edible products that may be used in numerous preparations and are easily found in markets or supermarkets. Furthermore, illegal commerce of these products in a rural context is a concrete possibility. For these reasons, determination of mycotoxins on animal organs, such as the liver and kidneys, is mandatory, and the development of adequate procedures for extraction and analytical techniques are highly desirable.

The aim of this paper was to develop and validate a useful method for determining the presence of ZON and metabolites in alternative biological matrices, represented by animal liver and kidney samples (chicken, pig, and lamb) to allow the exposure assessment of ZON, α-ZOL, β-ZOL, ZAN, α-ZAL and β-ZAL. In this work, a solid-liquid extraction procedure coupled with GC-MS/MS analysis was developed for direct determination of ZON and its main metabolites in liver and kidney samples easily obtained from Spanish markets. Critical factors that could affect the extraction efficiency were studied. The procedure was validated and used to quantify the concentrations of free and conjugated mycotoxins in different liver samples by using β-glucuronidase.

## 2. Results and Discussion

### 2.1. Validation of the Mycotoxin Determination in Animal Liver

A GC-MS/MS method was used for analysis of ZON, α-ZOL, β-ZOL, ZAN, α-ZAL and β-ZAL in pig, chicken, and lamb liver samples. Previous studies have used LC-MS for quantification of ZON and its metabolites in different tissues; however, GC-MS has been used for analysis of ZON in grains [29]. The concentrations of ZON and its metabolites in liver samples usually occur in units of µg/L; hence it is important to optimize the GC-MS/MS method to determine these possible levels. The suitability of the quantitation method for liver mycotoxin levels was evaluated by a validation study. The GC-MS/MS method was performed for linearity, matrix effect, accuracy, repeatability (intraday and interday precision) and sensitivity, following the EU Commission Decision 2002/657/EC [30]. 

The limit of detection (LD) and limit of quantification (LQ) were estimated for a signal-to-noise ratio (S/N) ≥ 3 and ≥10, respectively, from chromatograms of samples spiked at the lowest level validated. LD and LQ values were established as a mean of the LD and LQ for a mix with all studied matrices, taking into account the possible heterogeneity of the samples (Table 1). 

**Figure 1:**
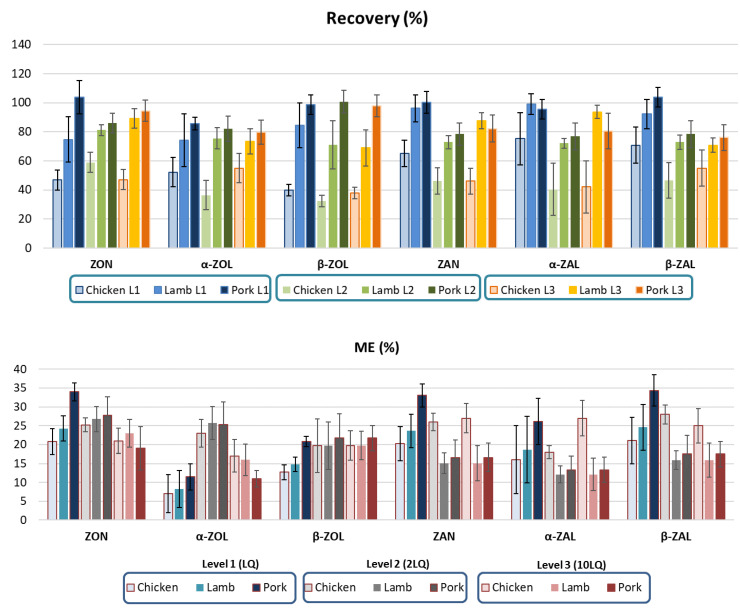
Accuracy (recovery), precision (bar borders RSD_R_) and matrix effect (ME) data study at three levels (L1: LQ; L2: 2LQ; L3: 10LQ) in studied livers (Zearalenone (ZON), zearalanone (ZAN) and their phase I metabolites: α-zearalenol (α-ZOL), β-zearalenol (β-ZOL), α-zearalalanol (α-ZAL) and β-zearalalanol (β-ZAL)).

Matrix-matched calibration curves were constructed at concentration levels between the LQ to 1 μg/mL. Correlation between the response and the amount of analytes was verified by plotting signal intensity against analyte concentrations. Good linearity was achieved in all cases with regression coefficients higher than 0.9997. Calibration curves were checked at the end of the analysis to assess the response drift of the method. The matrix effect (ME) was assessed for each analyte by comparing the slope of the standard calibration curve (standard) with that of the matrix-matched calibration curve (matrix), for the same concentration levels (Figure 1). The ME values were suppression signals and ranged between (11 ± 4)% and (27 ± 4)% for α-ZOL in pork liver and ZAN and α-ZAL in chicken liver, respectively. The accuracy of the studied mycotoxins extraction from liver samples was determined by a liver sample fortification procedure (Figure 1). The values of recovery ranged between (104 ± 7)% and (76 ± 9)% for β-ZAL in chicken liver and β-ZAL in pork liver, respectively. The blank was initially prepared and tested negative, and was fortified before the extraction procedure with three different mycotoxin levels at LQ, 2 LQ and 10 LQ (*n* = 6). Method precision was estimated by calculating the relative standard deviation (RSD_R_) using the results obtained during intra-day and inter-day replicates analysis (*n* = 9). The RSD_R_ values were bellow to 11% and proved good intra-day and inter-day precision.

### 2.2. Presence of Zearalenone (ZON), Zearalanone (ZAN) and Their Metabolites in Liver Samples 

The natural occurrence of six different mycotoxins (ZON, α-ZOL, β-ZOL, ZAN, α-ZAL and β-ZAL) was investigated in livers from chicken (*n* = 31), pig (*n* = 30) and lamb (*n* = 30). All samples were bought in different supermarkets from the Valencian Community in Spain during the period comprised between 2021 and 2022.

**Figure 2:**
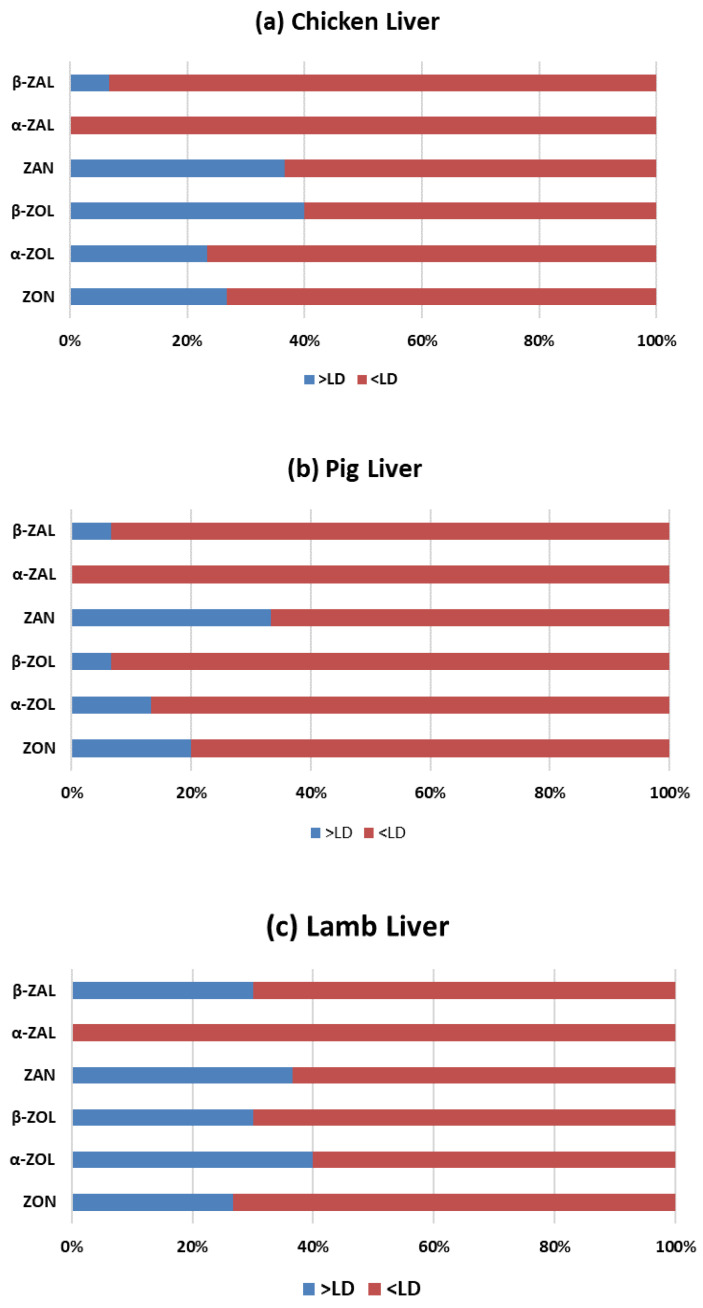
Frequency (%) of the studied mycotoxins and its metabolites in chicken (**a**), pig (**b**) and lamb (**c**) livers samples. (LD: limit of detection; Zearalenone (ZON), zearalanone (ZAN) and their phase I metabolites: α-zearalenol (α-ZOL), β-zearalenol (β-ZOL), α-zearalalanol (α-ZAL) and β-zearalalanol (β-ZAL).

Mycotoxins and metabolites detected in the analyzed liver samples are presented in Table 2. Results show that 63 out of 91 samples (69%) were contaminated with at least one mycotoxin or metabolite at variable levels. The most present mycotoxin by investigated animal samples was β-ZOL (42%) in chicken liver samples, followed by α-ZOL in lamb liver samples (39%), and ZAN for pig liver samples (33%) (Figure 2). The number of positive samples with one mycotoxin was 69% and α-ZAL values were below the sensitivity of the method. A total of 60%, 54% and 6% of the samples of pig, chicken, and lamb, respectively, were not positive for any mycotoxins. 

Regarding the animal tissue used for the analysis, the highest incidence of mycotoxins was associated with lamb liver samples (93%), followed by chicken (46%) and pig (40%). Concerning the ranges, the highest ranges were found in chicken liver samples (LD-152.62 ng/g), followed by lamb liver samples (LD-24.71 ng/g) and pig liver samples (LD-1.36 ng/g), with the highest levels of β-ZOL, β-ZAL and ZAN, respectively (Table 2).

α- and β-ZON are important and accurate markers for exposure to these mycotoxins and usually analyzed in urine, due to, their values change rapidly in urine [31]. The detected mycotoxins in animal livers indicate a chronic exposure to these mycotoxins and are a possible risk to the animal’s health due to the adverse effects that they can produce [32], specifically in pigs, the most sensitive animal species, in terms of the oestrogenic activity of zearalenone and its metabolites. Furthermore, Gajęcka et al., 2016 [33] reported that α-ZOL has a higher binding affinity to estrogen receptors than ZON [32]. 

#### 2.2.1. Zearalenone (ZON) and Its Metabolites Occurrence

The analytical data showed that the ZON metabolite with the highest incidence was β-ZOL for chicken liver samples, with 42% (13 out of 31 samples) at maximum levels of 152.62 ng/g, ZON for pig liver samples with 20% (6 out of 30 samples) at maximum levels of 0.13 ng/g and α-ZOL for lamb liver samples with 39% (12 out of 30 samples) at maximum levels of 23.81 ng/g. Levels detected for ZON, α-ZOL and β-ZOL were below 30.79 ng/g, 23.81 ng/g and 152.61 ng/g, respectively (Table 2). In cases where the samples were being cooked and eaten by consumers, the exposure would be small because if it is calculated as the daily intake, the value will be below the TDI reported by EFSA of 0.25 µg/kg bw for ZON [34].

In previous studies, very limited reports have been documented for the presence of ZON contaminating chicken, pig or lamb liver. However, Iqbal et al. (2014) [35] reported low levels of ZON at 2.97 µg/kg, 4.91 µg/kg and 5.10 µg/kg in domestic chicken liver, boiler breed chicken liver, and layer breed chicken liver, respectively. Other studies reported the presence of ZON in the bile of breeding cows with a contamination rate of 96.2% [36]. 

Liver and enterocytes play an important role in ZON metabolism; in fact, it varies depending on the animal. This variation may be related to hepatic biotransformation. The literature reveals that in guinea pigs, both α-ZOL and β-ZOL were formed in equal amounts [37]; in pigs, α-ZOL is formed in a higher amount compared to β-ZOL; whereas, in chicken, β-ZOL is produced in high quantities by the hepatic microsomes [37,38]. According to these studies, our results in chicken livers showed higher amounts of β-ZOL (22.73 ± 55.0 ng/g) than α-ZOL (7.46 ± 9.37 ng/g). It is important to note that β-ZOL presented higher potential estrogenic amounts than ZON and α-ZOL [2].

#### 2.2.2. Zearalanone (ZAN) and Its Metabolites Occurrence

The highest incidence was for ZAN for all liver samples: 12 out of 31 chicken liver samples (39%) (max. 7.92 ng/g), 11 out 30 lamb liver samples (37%) (max. 4.94 ng/g)) and 10 out of 30 pig liver samples (33%) (max. 1.36 ng/g) (Table 2).

α-ZAL, a resorcyl lactone, was not detected in any of the samples analyzed, and these results are in agreement with the literature [31]. It is important to remar, that α-ZAL was used as a growth promoter in the United States many years ago but nowadays it is banned [39]. 

Other authors have detected smaller quantities for α-ZAL than the rest of analyzed zearalenones. Döll et al. (2003) reveal that in piglets’ liver, ZAN, α-ZAL and β-ZAL were below 100, 50 and 200 ng/g, respectively [40]. Moreover, it has been shown that in the enzymatic reduction of ZAN, smaller amounts of zearalenols are produced. Malekinejad et al. (2006) [37] reported differences between mammalian species in the hepatic transformation of ZAN to its reduced and glucuronide metabolites. All these mammalian species converted large percentages of ZAN and metabolites to the corresponding glucuronides [37]. On the other hand, the comparison between species suggests that pigs, which preferentially produce α-ZAL over the β analogue by five-fold, are predicted to be more sensitive to the oestrogenic effects of ZAN than other animal species [37].

In others studies, few levels of α-ZAL were observed in pig; indeed, a significant fraction of ZAN was found in the form of α-ZAL and its respective glucuronide conjugates [41], while cows converted ZAN predominantly to β-ZAL [42]. Smaller amounts of further reduced metabolites (i.e., α- and β-ZAL) were observed in other ruminant species [31]. However, ovine metabolism of ZON produces at least five compounds, including α- and β-ZOL, α- and β-ZAL, and ZAN [43].

#### 2.2.3. Simultaneous Presence of Analyzed Mycotoxins and Metabolites

The natural copresence of analyzed mycotoxins was evaluated in all animal livers bought in the Valencian Community (Spain) in order to have an approximation of the oral exposure of these animals. Previous studies indicate that ZON is usually found to co-occurrence with its metabolites [19].

**Figure 3:**
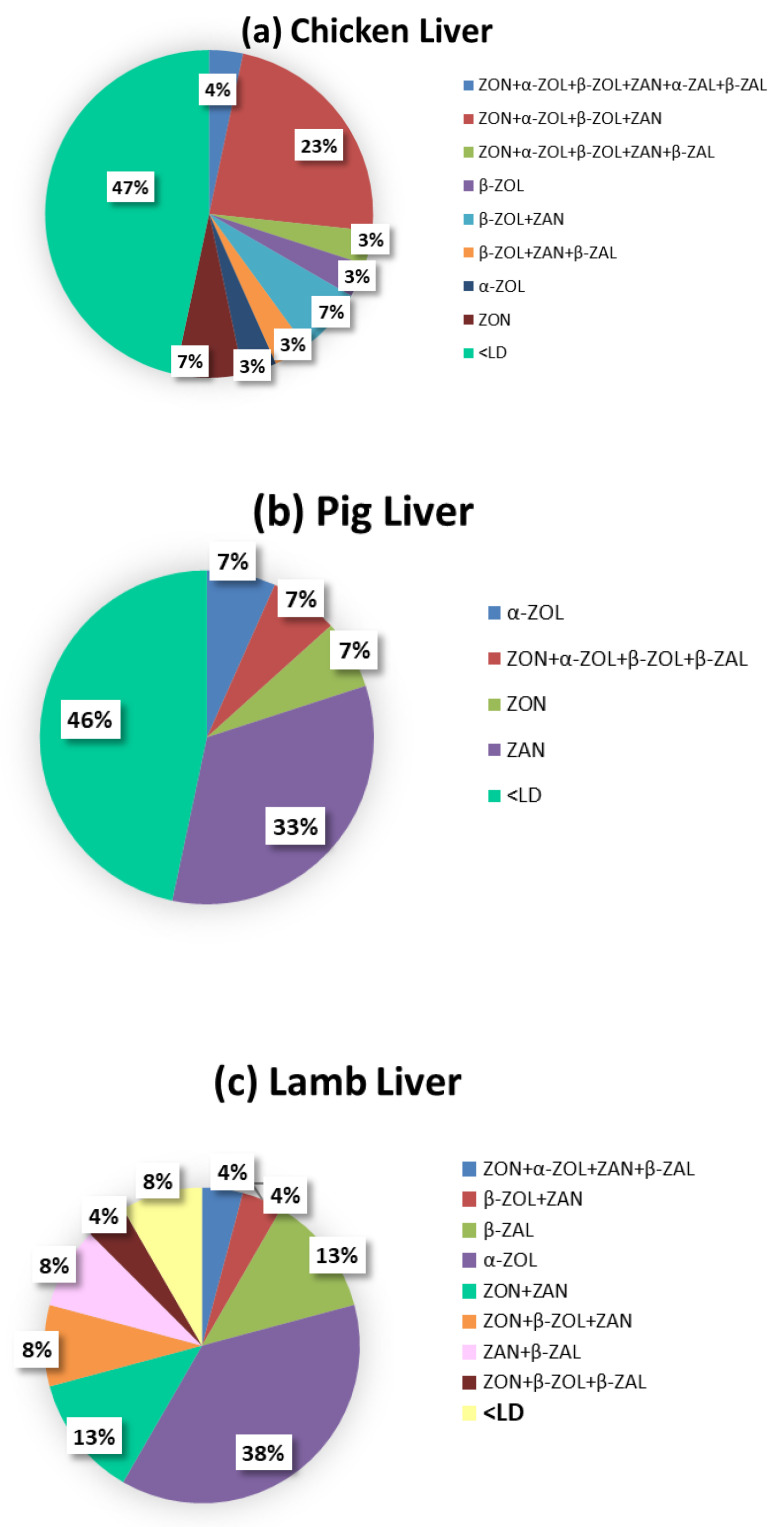
Co-occurrence of mycotoxins and metabolites in the three of analyzed animal liver samples (**a**) chicken, (**b**) pig and (**c**) lamb. (Zearalenone (ZON), zearalanone (ZAN) and their phase I metabolites: α-zearalenol (α-ZOL), β-zearalenol (β-ZOL), α-zearalalanol (α-ZAL) and β-zearalalanol (β-ZAL)).

All in all, from a descriptive standpoint, a co-occurence of different metabolites was found in 30% of analyzed samples. The results show a combination of five mycotoxins in 2.2% different samples (ZON + α-ZOL + β-ZOL + ZAN + β-ZAL) (Figure 3). While, four and three associations were also observed in 8.8% (ZON + α-ZOL + β-ZOL + ZAN and ZON + α-ZOL + ZAN + β-ZAL) and 5.5% samples (β-ZOL + ZAN + β-ZAL, ZON + β-ZOL + β-ZAL and ZON + β-ZOL + ZAN), respectively.

It was also observed that 39%, 7%, and 47% of chicken, pig, and lamb liver samples, respectively, were contaminated by at least two toxins. The lowest co-occurrence frequency was found in pig liver samples with 7% of samples contaminated with four mycotoxins (ZON + α-ZOL + β-ZOL + β-ZAL). In chicken liver samples, 16% showed the combination ZON + α-ZOL + β-ZOL + ZAN, followed by 13% β-ZOL + ZAN, 7% ZON + α-ZOL + β-ZOL + ZAN + β-ZAL and 3% β-ZOL + ZAN + β-ZAL. A total of 13% of samples showed only one mycotoxin or metabolite and 48% of the samples were <LQ. Lamb liver samples showed the highest binary combinations (30%): ZON + ZAN (10%), β-ZOL + ZAN (10%), ZAN + β-ZAL (7%) and β-ZOL + β-ZAL (3%). Only four samples (13%) showed the combination of three mycotoxins (ZON + β-ZOL + ZAN and ZON + β-ZOL + β-ZAL) and one sample (3%) a combination of four mycotoxins (ZON + α-ZOL + ZAN + β-ZAL). A total of 47% of the samples did not show a co-occurrence and 7% of the samples were <LQ. Lastly, pig liver samples had the lowest co-occurrence since only 7% of the samples (2 out of 30 samples) were contaminated by more than one mycotoxin or metabolite (ZON + α-ZOL+β-ZOL + β-ZAL). A total of 53% of the samples did not show co-occurrence and 40% of the samples were <LQ.

## 3. Conclusions

The validated GC-MS/MS method presented good results in terms of accuracy, sensitivity and robustness for the simultaneous determination of six target mycotoxins in three different type of animal liver (chicken, lamb and pig). The method was suitable for analyzing 91 animal liver samples. The analytical data showed that 69% of analyzed liver samples were contaminated with at least one of the analyzed mycotoxins. β-ZOL was the most detected (42%), with the highest value (max. 152.62 ng/g) observed in chicken liver samples. This can be associated with the fact that chickens are mainly fed with corn or feed rich in corn, and this cereal is the ideal substrate for grown *Fusarium graminearum*, which is the primary producer of ZON [5,7,15,40,44]. In this sense, the implementation of a hazard analysis and critical control point (HACCP) system should be applied throughout the food chain from primary production to final consumer to reduce the presence and production of mycotoxins in feed. Furthermore, control systems to analyze and monitor mycotoxins should be strengthened to prevent exposure and protect animal and human health.

## 4. Material and Methods

### 4.1. Standards

Mycotoxin standards and metabolites specifically ZON, α-ZOL, β-ZOL, ZAN, α-ZAL and β-ZAL were obtained from Sigma-Aldrich (St. Louis, MO, USA). Individual stock solutions of all analytes were prepared at identical concentration (1000 mg/L) in methanol. The stock solutions were diluted with acetonitrile to obtain a working standard solutions of 50 mg/L with the six mycotoxins. All standards were stored in darkness and kept at −20 °C until the GC-MS/MS analysis.

### 4.2. Chemical, Reagents and Other Material

The derivatization reagent composed of BSA (N,O-bis(trimethylsilyl)acetamide) + TMCS (trimethylchlorosilane) + TMSI (N-trimethylsilyimidazole) (3:2:3) was purchased from Supelco (Bellefonte, PA, USA).

Sodium dihydrogen phosphate and disodium hydrogen phosphate, used to prepare phosphate buffer, were acquired from Panreac Quimica S.L.U. (Barcelona, Spain). β-Glucuronidase Type H-1 from Helix pomatia (glucuronidase activity: ≥300,000 units/g solid and sulfatase activity: ≥10,000 units/g solid) was purchased from Sigma–Aldrich (St. Louis, MO, USA).

All solvents, acetonitrile, hexane and methanol (HPLC grade) were purchased from Merck KGaA (Darmstadt, Germany). Anhydrous magnesium sulfate (thin powder) was obtained from Alfa Aesar GmbH & Co (Karlsruhe, Germany); sodium chloride was purchased from Merck and C18-E (50 μm, 65A) was purchased from Phenomenex (Torrance, CA, USA).

### 4.3. Apparatus

ZON, α-ZOL, β-ZOL, ZAN, α-ZAL and β-ZAL were analyzed by GC-MS/MS. Aliquots of 1 μL of the derivatized extract were injected in splitless mode at 250 °C in programmable temperature vaporization (PTV). A GC system Agilent 7890A coupled with an Agilent 7000A triple quadrupole mass spectrometer with inert electron-impact ion source and an Agilent 7693 autosampler (Agilent Technologies, Palo Alto, CA, USA) were used for MS/MS analysis [44]. An HP-5 MS 30 m × 0.25 mm × 0.25 μm capillary column was used. All analytes eluted within 17 min, reaching the requirement for a high throughout determination.

The MS was operating in electron impact ionization (EI, 70 eV). The source and transfer line temperatures were 230 °C and 280 °C, respectively. The collision gas for MS/MS experiments was nitrogen, and the helium was used as carrier gas at a fixed pressure of 20.3 psi, both at 99.999% purity supplied by Carburos Metálicos S.L. (Barcelona, Spain). The oven temperature program was initially 80 °C, and the temperature increased to 245 °C at 60 °C/min. After a 3-min hold time, the temperature was increased to 260 °C progressively at 3 °C/min and finally to 270 °C at 10 °C/min and then held for 10 min. The analysis was performed with a solvent delay of 3 min in order to prevent instrument damage.

Quantitation data were acquired at SRM mode, with a couple of transition ions, and the mass spectrometer operated in electrospray ionization (EI) mode (Table 1). The transfer line and source temperatures were 280 °C and 230 °C, respectively. The EI energy used was 70 eV as in that region the maximum abundance was observed. The collision energies varied from 5 to 20 eV, depending on the precursor and product ions. The analysis was performed with a filament-multiplier delay of 3.50 min. The collision gas for MS/MS experiments was nitrogen, and the helium was used as quenching gas, both at 99.999% purity supplied by Carburos Metálicos S.L. (Barcelona, Spain). The dwell times also varied from 5 to 35 eV. Data was acquired and processed using the Agilent Masshunter version B.04.00 software.

### 4.4. Sample Preparation

Livers were analyzed for total ZON, α-ZOL, β-ZOL, ZAN, α-ZAL and β-ZAL concentrations, including their conjugated glucuronides.

For the β-Glucuronidase hydrolysis, the enzymatic hydrolysis method used to de-conjugate glucuronides was adapted from [45]. Each sub-sample of tissue (0.5 g) was homogenized in 0.64 mL pH 5.0 ammonium acetate buffer (1 M, BioWorld, Fisher Scientific, 1768, Pittsburgh, PA, USA). Homogenization was carried out using a Polytron PT 10–35 with PTA-10T generator (Kinematica AG, Luzern, Switzerland). After homogenization, 10 μL of β-glucuronidase (Helix pomatia, H-2, Millipore Sigma, Saint Louis, MO, USA) containing 100,000 U of β-glucuronidase/mL was added to the mixture, incubated at 37 °C for 18 h, then allowed to cool to room temperature. Methods for extraction of ZEN and α-ZEL from enzyme-hydrolyzed liver tissue were adapted from standard mycotoxin analysis methods previously described by Mahmoud et al. (2018) [46].

Then, 1.5 mL of acetonitrile was added to 0.5 g of liver and vortexed for 1 min. It was sonicated 10 min at room temperature and centrifuged at 4000 rpm for 3 min at 5 °C. Supernatant was collected in a 15 mL falcon and 150 mg of MgSO_4_ and 50 mg of C18 were added prior to be vortexed for 1 min, sonicated 10 min at room temperature and centrifuged at 4000 rpm for 3 min at 5 °C. Then, the upper layer was collected in a vial and it was evaporated to dryness under nitrogen flow.

The dry extract was derivatized with 50 mL of BSA + TMCS + TMSI (3:2:3) and left for 30 min at room temperature. After that, it was diluted to 200 µL with hexane and mixed thoroughly on a vortex for 30 s. Then, the diluted derivatized sample was added with 1 mL of phosphate buffer (60 mM, pH 7) to purify the derivate with a liquid–liquid extraction and the upper layer (hexane phase) was transferred to an autosampler vial for the chromatographic analysis.

### 4.5. Sampling

Livers (*n* = 91) were purchased from different supermarkets of the Valencian Community (Spain) during the period comprised between October 2021 and February 2022. Chicken liver (*n* = 31), pork liver (*n* = 30) and lamb liver (*n* = 30) were collected and milled separately. Then, 0.5 g of each sample were weighted and kept at −20 °C in dark and dry place until further analysis.

### 4.6. Method Validation

Commission Decision 2002/657/EC [30] was used as guidelines for the validation studies. All the parameters were evaluated by spiking blank samples. For identification purposes, retention times of mycotoxins in standards and samples were compared at tolerance of ±0.5%. Moreover, in accordance with the 2002/657/EC Decision [30], the relative ion intensity of analytes studied in the standard solution and the spiked samples at the concentration levels used for the calibration curve were compared.

Method performance characteristics such as linearity, LD, LQ, matrix effect, extraction recovery, repeatability and reproducibility were evaluated for all tested mycotoxins.

## Figures and Tables

**Table 1:** Quantification and confirmation transitions, retention time (Rt) and limits of quantification (LQ) and detection (LD) of the analyzed mycotoxins.

Mycotoxin *	Rt (min)	Quantification Transition (m/z)	Confirmation Transition (m/z)	LD (ng/g)	LQ (ng/g)
ZON	16.77	462 > 151	462 > 151	0.061	1.25
α-ZOL	16.74	305 > 289	305 > 289	0.031	0.31
β-ZOL	15.83	536 > 446	536 > 446	0.118	0.63
ZAN	15.84	449 > 335	449 > 335	0.017	0.31
α-ZAL	15.84	433 > 309	433 > 309	0.239	2.50
β-ZAL	15.89	307 > 292	307 > 292	0.361	1.25

* Zearalenone (ZON), zearalanone (ZAN) and their phase I metabolites: α-zearalenol (α-ZOL), β-zearalenol (β-ZOL), α-zearalalanol (α-ZAL) and β-zearalalanol (β-ZAL).

**Table 2:** Incidence (I), mean (M ± SD), mean of positive samples (Mp ± SD) and range results of detected mycotoxins in analyzed liver samples.

	Chicken Liver (*n* = 31)				Pig Liver (*n* = 30)				Lamb Liver (*n* = 30)			
Analyte *	I	M ± SD (ng/g)	Mp ± SD (ng/g)	Range (ng/g)	I	M ± SD (ng/g)	Mp ± SD (ng/g)	Range (ng/g)	I	M ± SD (ng/g)	Mp ± SD (ng/g)	Range (ng/g)
ZON	9	1.95 ± 5.96	8.94 ± 13.15	0.09–30.79	6	0.02 ± 0.04	0.13 ± 0.02	0.09–0.13	8	0.82 ±1.51	4.08 ± 1.70	1.94–5.91
α-ZOL	8	1.44 ± 4.21	7.46 ± 9.37	0.11–21.50	4	0.05 ± 0.16	0.48 ± 0.43	0.11–0.83	12	6.19 ± 9.16	20.64 ± 10.68	6.62–23.81
β-ZOL	13	7.15 ± 27.46	22.73 ± 55.01	0.25–152.62	2	0.02 ± 0.10	0.49 ± 0.11	0.31–0.43	9	0.58 ± 1.47	2.60 ± 2.94	0.19–4.97
ZAN	12	2.85 ± 7.92	9.83 ± 15.48	0.78–43.33	10	0.29 ± 0.43	1.17 ± 0.26	0.75–1.36	11	1.03 ± 1.49	3.75 ± 1.31	1.50–4.94
β-ZAL	3	1.49 ± 6.21	20.54 ± 21.38	6.01–33.92	2	0.04 ± 0.19	0.87 ± 0.66	0.30–1.00	9	5.61 ± 8.96	24.92 ± 5.33	15.33–24.71

* Zearalenone (ZON), zearalanone (ZAN) and their phase I metabolites: α-zearalenol (α-ZOL), β-zearalenol (β-ZOL), α-zearalalanol (α-ZAL) and β-zearalalanol (β-ZAL); *n*: number of samples analysed.

## Data Availability

Not applicable.
